# Virulence of entomopathogenic fungi against fall armyworm, *Spodoptera frugiperda* (Lepidoptera: Noctuidae) under laboratory conditions

**DOI:** 10.3389/fphys.2023.1107434

**Published:** 2023-03-08

**Authors:** Atif Idrees, Ayesha Afzal, Ziyad Abdul Qadir, Jun Li

**Affiliations:** ^1^ Guangdong Key Laboratory of Animal Conservation and Resource Utilization, Guangdong Public Laboratory of Wild Animal Conservation and Utilization, Institute of Zoology, Guangdong Academy of Sciences, Guangzhou, China; ^2^ Guizhou Provincial Key Laboratory for Agricultural Pest Management of the Mountainous Region, Scientific Observing and Experimental Station of Crop Pest in Guiyang, Institute of Entomology, Ministry of Agriculture, Guizhou University, Guiyang, China; ^3^ Institute of Molecular Biology and Biotechnology, The University of Lahore, Lahore, Pakistan; ^4^ Honeybee Research Institute, National Agricultural Research Centre, Islamabad, Pakistan; ^5^ Department of Entomology and Wildlife Ecology, University of Delaware, Newark, DE, United States

**Keywords:** fall army worm, egg mortality, neonate mortality, feeding performance, entomopathogenic fungi

## Abstract

Maize is an essential crop of China. The recent invasion of *Spodoptera frugiperda*, also known as fall armyworm (FAW), poses a danger to the country’s ability to maintain a sustainable level of productivity from this core crop. Entomopathogenic fungi (EPF) *Metarhizium anisopliae* MA, *Penicillium citrinum* CTD-28 and CTD-2, *Cladosporium* sp. BM-8, *Aspergillus* sp. SE-25 and SE-5, *Metarhizium sp.* CA-7, and *Syncephalastrum racemosum* SR-23 were tested to determine their effectiveness in causing mortality in second instars, eggs, and neonate larvae. *Metarhizium anisopliae* MA, *P. citrinum* CTD-28, and *Cladosporium* sp. BM-8 caused the highest levels of egg mortality, with 86.0, 75.3, and 70.0%, respectively, followed by *Penicillium* sp. CTD-2 (60.0%). Additionally, *M. anisopliae* MA caused the highest neonatal mortality of 57.1%, followed by P. *citrinum* CTD-28 (40.7%). In addition, *M. anisopliae* MA, *P. citrinum* CTD-28, and *Penicillium* sp. CTD-2 decreased the feeding efficacy of second instar larvae of FAW by 77.8, 75.0, and 68.1%, respectively, followed by *Cladosporium* sp. BM-8 (59.7%). It is possible that EPF will play an important role as microbial agents against FAW after further research is conducted on the effectiveness of these EPF in the field.

## 1 Introduction

China’s reliance on maize production is evidenced by the fact that the nation now ranks among the world’s top net importers of grain (Tong, 2000). China is the world’s second-largest producer of corn, behind only the United States. The fall armyworm (FAW), also known as *Spodoptera frugiperda*, J. E. Smith, 1797 (Lepidoptera: Noctuidae), is regarded as a serious and devastating pest of maize. Because of this, maize production continues to be at risk. The American continent, especially its tropical and subtropical parts, is the origin of this pest (Sparks, 1979); it has recently attacked several countries and its presence has had a profoundly negative influence on world food security in all of the locations that it has colonized. FAW was firstly seen in Nigeria and Ghana (Goergen et al., 2016), but it quickly migrates in other African countries (Stokstad, 2017). In 2018, FAW was reported in the state of Karnataka, which is located in the southern portion of India (Sharanabasappa et al., 2018). By the end of 2018, its occurrence was also documented in nations located in Southeast Asia, including Thailand, Bangladesh, and Myanmar (Guo et al., 2018).

The corn strain of FAW was firstly observed in China in December 2018 (Zhang et al., 2019; Sun et al., 2021). Major provinces like Yunnan, were severely attacked by this invasive pest (Zhang et al., 2021), which damages important staple and economical crops but preferably feeds on corn and sorghum in China (Montezano et al., 2018). Significant damage was observed in cornfields of Yunnan during the initial attack of FAW (Yang et al., 2019). The damage losses to the maize crop were up to 22, 67, 32% and 47% in Ghana, Zambia, Ethiopia, and Kenya, respectively (Day et al., 2017; Kumela et al., 2019). Hence, this invasive pest is responsible for causing financial losses of up to 4.66 billion USD dollars in Africa (Rwomushana et al., 2018). FAW causes reduction in yield of 78, 80% and 90% of peanut, barley, and wheat crops, respectively (He et al., 2020; Yang et al., 2020). FAW also causes damage to tobacco crops if its population peaks (Xu et al., 2019).

Recently, the development of biopesticides, including plant extracts and novel insecticides is the serious concern of researchers for reducing crop damage and maximizing agricultural yield (Cook et al., 2004; Idrees et al., 2016; Idrees et al., 2017; Luo et al., 2018; Qadir et al., 2021; Ahmed et al., 2022; Liu et al., 2022; Idrees et al., 2022b). However, regular use of pesticides to control FAW have become a regular practice which is harmful to the ecosystem and natural enemies (Gu et al., 2018; Cai et al., 2017).

The application of entomopathogenic fungi (EPF) is considered to be one of the most common strategies for the control of FAW (Idrees et al., 2021; Idrees et al., 2022a). Because EPF and synthetic insecticides have distinct modes of action, therefore, EPF do not act as fast as synthetic insecticides to kill insect pests (Hajek, 1989; Fargues et al., 1994). EPF can cause infection when spores come in contact with the arthropod host. Fungal spores germinate and breach the insect cuticle through enzymatic degradation and mechanical pressure to gain entry into the insect body under an ideal condition. The EPF have fast multiplication after invading the insect tissues, and emerge from the dead insect to produce more fungal spores (Dara, 2017; Altinok et al., 2019; Ebani and Mancianti, 2021). However, it is worth noting that these EPF serve to minimize crop damage by causing host pest infection, which leads to a decrease in feeding, egg laying, development, and mating and disturbs the physiological function of pests (Thomas et al., 1997). Isolates of EPF exhibited considerable mortality of eggs and neonate larvae of FAW (Akutse et al., 2019) and significantly reduced the feeding efficacy of larvae (Qadir et al., 2021; Idrees et al., 2022a).

A few studies have been performed on the effectiveness of native EPF for the management of FAW in China. Therefore, the purpose of the present research was to evaluate the virulence of EPF against immature stages (eggs, neonate larvae and pupae) as well as on the feeding performance of FAW larvae to develop microbial-based biopesticides at the commercial level against FAW.

## 2 Materials and methods

### 2.1 Insect rearing

Eggs of FAW were collected from an established colony in the laboratory. The eggs were kept in a ventilated rectangular plastic box (28 cm^3^ × 17 cm^3^ × 18 cm^3^). The neonate larvae were fed with fresh maize insecticides free leaves. The first^−^ to third^-^instar larvae were kept in a rectangular plastic box (28 cm^3^ × 17 cm^3^ × 18 cm^3^), while fourth^−^ to sixth-instar larvae were separately placed in six-well plates to prevent cannibalism until pupation. The new emerging adults were placed in cylindrical glasses. A paper towel was used to cover the top portion of the adult glasses, and sterile cotton balls were placed inside a plastic bottle lid soaked with a 10% concentration of honey. The larvae were kept at 25°C ± 2°C, with a photoperiod of 12:12 (dark: light) and 65% ± 5% relative humidity (RH). Fifty laboratory-reared generations of larvae were used in the present study.

### 2.2 Entomopathogenic fungal Isolates

The EPF, *Metarhizium anisopliae* MA, *Penicillium citrinum* CTD-28 and CTD-2, *Cladosporium* sp. BM-8, *Aspergillus* sp. SE-25 and SE-5, *Metarhizium* sp. CA-7, and *Syncephalastrum racemosum* SR-23 were evaluated against immature stages and feeding efficacy of FAW. Detailed information about EPF species is described in Table 1. EPF species were obtained from laboratory collection at IZ-GDAS.

**TABLE 1:** Information about the fungal isolates evaluated in the present study for the management of fall armyworm.

Fungal species	Isolates	Host or source of origin	Site of origin (Country)	Year of isolation
*Metarhizium anisopliae*	MA	*Spodoptera frugiperda* (Lepidoptera: Noctuidae)	Guangzhou, Guangdong	2019
*Penicillium citrinum*	CTD-28	*Spodoptera frugiperda* (Lepidoptera: Noctuidae)	Guangzhou, Guangdong	2019
*Penicillium* sp	CTD-2	*Spodoptera frugiperda* (Lepidoptera: Noctuidae)	Guangzhou, Guangdong	2019
*Cladosporium* sp	BM-8	*Spodoptera frugiperda* (Lepidoptera: Noctuidae)	Guangzhou, Guangdong	2019
*Aspergillus versicolor*	SE-25	*Trachymela sloanei* (Lepidoptera: Noctuidae)	Shenzhen, Guangdong	2019
*Aspergillus* sp	SE-5	*Trachymela sloanei* (Lepidoptera: Noctuidae)	Shenzhen, Guangdong	2019
*Metarhizium* sp	CA-7	*Trachymela sloanei* (Lepidoptera: Noctuidae)	Shenzhen, Guangdong	2019
*Syncephalastrum racemosum*	SR-13	*Trachymela sloanei* (Lepidoptera: Noctuidae)	Shenzhen, Guangdong	2019

The fungal isolates were cultured by spreading a small portion of it on Sabouraud dextrose agar media (inoculation) in Petri dished (90 mm in diameter). The Petri dishes were incubated in dark incubator for 2–3 weeks. Fungal conidia were harvested from 2- to 3-week-old sporulated cultures and suspended in 10 mL of distilled water with 0.05% Tween-80 in universal bottles containing glass beads containing six to nine beads (3 mm in diameter) for each bottle. The fungal conidial suspensions were vortexed for 5 minutes at approximately 700 rpm to break up the conidial clumps and verify that the suspension was homogenous. Three conidial concentrations (1 × 10^6^, 1 × 10^7^, and 1 × 10^8^ conidia/mL) were adjusted using hemocytometer before the bioassay.

The viability tests of fungal isolates were further conducted prior to starting the bioassay (Opisa et al., 2018). The eight fungal isolates showed ≥90% germination rates (Supplementary Table S1).

### 2.3 Efficacy of fungal Isolates on eggs and neonate larvae of FAW

FAW eggs that were one to 2 days old were taken from adult cylindrical glass. Under a light microscope, 50 eggs were divided using a camel hairbrush. A volume of 10 mL of each concentration was sprayed on a batch of 50 eggs using a manually atomized spray bottle (20 mL). A sterile paper towel was placed at the bottom of rectangular box to absorb the extra spore suspension. Sterilized distilled water with 0.05% Tween-80 was considered as a control. The eggs were then air-dried for an hour in a laminar flow hood after exposure to the treatment. The eggs were then put into Petri dishes and incubated at room temperature (25°C ± 2°C) with 65% ± 5% relative humidity (RH). Egg mortality were measured 7 days post treatment. Neonates larvae that emerged from the treated eggs were kept in a perforated rectangular plastic box coated with wet filter paper. Fresh maize leaves were provided to the neonate’s larvae daily. The data of neonate larvae were measured daily up to 14 days post treatment. The whole bioassays were repeated twice and completely randomized design (CRD) was used with three replications for each treatment.

The mortality of the neonate larvae was observed daily for 7 days. The cumulative mortality of both neonates and larvae of FAW was calculated by counting the dead eggs and neonates’ larvae of FAW divided by a total number of eggs at 14 days post-treatment (Idrees et al., 2022a). The cadavers were examined to see whether mycosis was present by the approach of (Aktuse et al., 2019). The dead cadavers were surface sterilized with alcohol (70%) and rinsed three times in a distilled water. The surface-sterilized dead cadavers were kept in Petri dishes containing sterile filter paper. The mortality due to target EPF was tested by observing hyphae and conidia on the body of dead cadavers.

### 2.4 Efficacy of fungal Isolates on second Instar larvae of FAW

For laboratory bioassays involving EPF evaluations against lepidopterous pests, early second^−^ or third-instar larvae are usually used because these larvae are easy to handle or manipulate during experimentation and are more susceptible to different insecticidal treatments than the other larval instars. In fact, first instar larvae are delicate and soft and are vulnerable to mechanical damage while manipulating or handling, while later (fourth - sixth) instar larvae are somewhat resistant and do not respond well to treatments. Therefore, only early second instar larvae were used in this study.

A group of 30 s instar larvae were transferred on fresh maize leaves unexposed to insecticides in a rectangular plastic box (28 cm^3^ × 17 cm^3^ × 18 cm^3^) covered with perforated lid. The bioassays were laid out according to CRD with three replications for each treatment. Afterwards, 10 mL of each concentration was sprayed on the larvae in each rectangular plastic container using an atomized manual spray bottle (20 mL). Each concentration was sprayed on every second instar larva to ensure that none escaped from the fungal spore suspension by hiding beneath the leaves’ surfaces. A sterile paper towel was put underneath the leaves to absorb the extra fungal suspension. The control larvae were treated with distilled water (0.05% Tween-80). Fresh maize leaves were given daily, and the treated larvae were kept at a temperature of 25°C ± 2°C. Larval mortality was recorded daily for up to 7 days. Mycosis was performed for the dead cadavers by the approach of Akutse et al. (2019).

### 2.5 Efficacy of fungal Isolates on the feeding performance of second Instar larvae of FAW

Using a hand-held atomizer spray bottle (20 mL), 15 FAW second-instar larvae were exposed to 10 mL of an EPF at three different concentrations (Fargues and Maniania, 1992). In a square plastic container, we inserted 12 g of fresh maize leaves. Sterilized distilled water with 0.05% Tween-80 was used to treat the control. The second instar larvae were weighed before and after being given a diet of fresh maize leaves. For this study, we calculated the feeding performance of the larvae in terms of feeding % by dividing the total weight of fresh maize leaf surface in Gram fed to the larvae by the total weight of leaves not eaten by the larvae and then multiplying that number by 100. The feeding performance was observed 24 and 48 h post treatment. The experiment was laid out according to CRD with three replications.

### 2.6 Efficacy of EPF on pupae of FAW

Using manual atomizer spray bottles (20 mL), 10 mL of each concentration of the investigated fungal isolates were applied to the pupae of FAW. Each treatment’s 15 pupae were put inside a 28 cm^3^ × 17 cm^3^ × 18 cm^3^ rectangular plastic box. Sterilized distilled water containing 0.05% Tween-80 was used to treat the control. Pupal mortality was observed for 15 days (Liu et al., 2022). The pupa did not turn black or emerge, or not show any movement upon touch considered as dead. The experiment was laid out according to CRD with three replications.

### 2.7 Statistical analysis

The Shapiro–Wilk test was used to analyze the normality of all of the stages before they were subjected to a one-way analysis of variance (ANOVA) using Tukey’s highly significant difference (HSD) *post hoc* test at a 95% level of significance (Shapiro and Wilk, 1965). In addition to graphical depiction, Statistix^®^ Version 8.1 was used to statistically evaluate the data (Analytical Software, Tallahassee, FL). Factorial ANOVA was used to analyze the interaction of the various factors, including the fungal conidial concentrations, treatments and immature stages, and this was followed by Tukey’s highly significant difference (HSD) *post hoc* test. SPSS (version 22.0) was used to perform for the data analysis.

## 3 Results

### 3.1 Effect of fungal Isolates on Eggs of FAW

**FIGURE 1:**
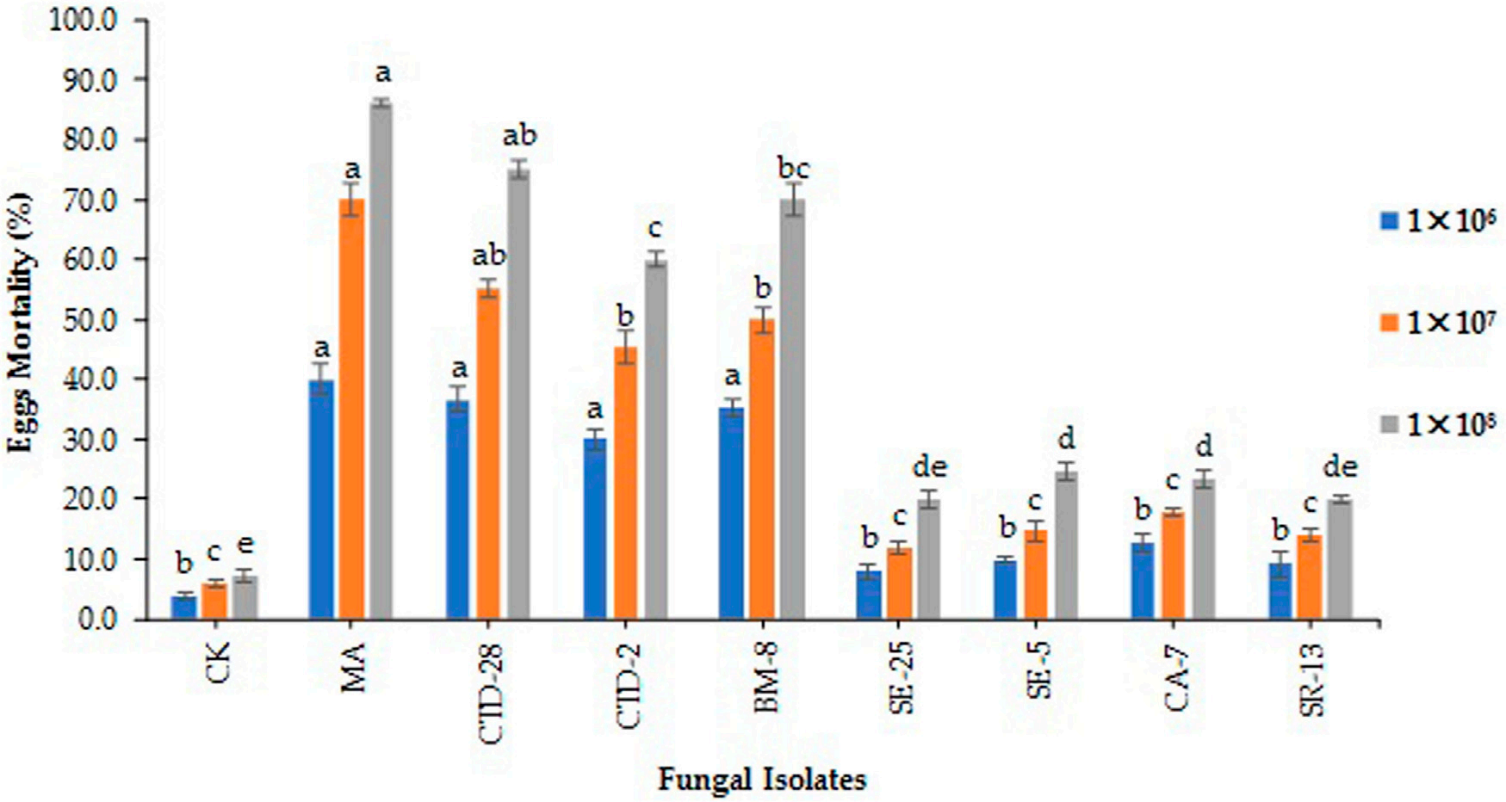
Fungal isolates induced fall armyworm’s egg mortality treated with different concentrations at 7 days post treatment. Error bars denote the mean ± standard error at the 95% confidence interval. Means followed by the same letters are not significantly different by Tukey’s test at *p* < 0.05.

The results revealed that the isolate of *M. anisopliae* MA caused 40% egg mortality, followed by the isolates of *P. citrinum* CTD-28 and *Cladosporium* sp. BM-8 which caused 36.7% and 35.3% egg mortality, respectively, treated with 1 × 10^6^ conidia/mL at 7 days post treatment (F_8, 18_ = 19.3; *p* = 0.0000). The isolate of *Penicillium* sp. CTD-2 induced 30.0% egg mortality compared to the control with 4.0% (Figure 1; Supplementary Table S2). The isolate of *M. anisopliae* MA caused the highest egg mortality of 70%, followed by the isolates of *P. citrinum* CTD-28 and *Cladosporium* sp. BM-8 which caused 55.3% and 50.0% egg mortality, respectively, treated with 1 × 10^7^ conidia/mL (F_8, 18_ = 44.1; *p* = 0.0000). The isolate of *Penicillium* sp. CTD-2 induced 45.3% egg mortality compared to 4.0% in the control (Figure 1; Supplementary Table S3). The isolates of *M. anisopliae* MA and *P. citrinum* CTD-28 caused 86.0% and 75.3% egg mortality, respectively, followed by *Cladosporium* sp. BM-8 and *Penicillium* sp. CTD-2 isolates which caused 70.0% and 60.0% egg mortality, respectively, treated with 1 × 10^8^ conidia/mL (F_8, 18_ = 99.1; *p* = 0.0000). The lowest egg mortality (24.7%) caused by the isolate of *Aspergillus* sp. SE-5 over the control with 7.3% (Figure 1; Supplementary Table S4).

### 3.2 Effect of fungal Isolates against neonate larvae of FAW

**FIGURE 2:**
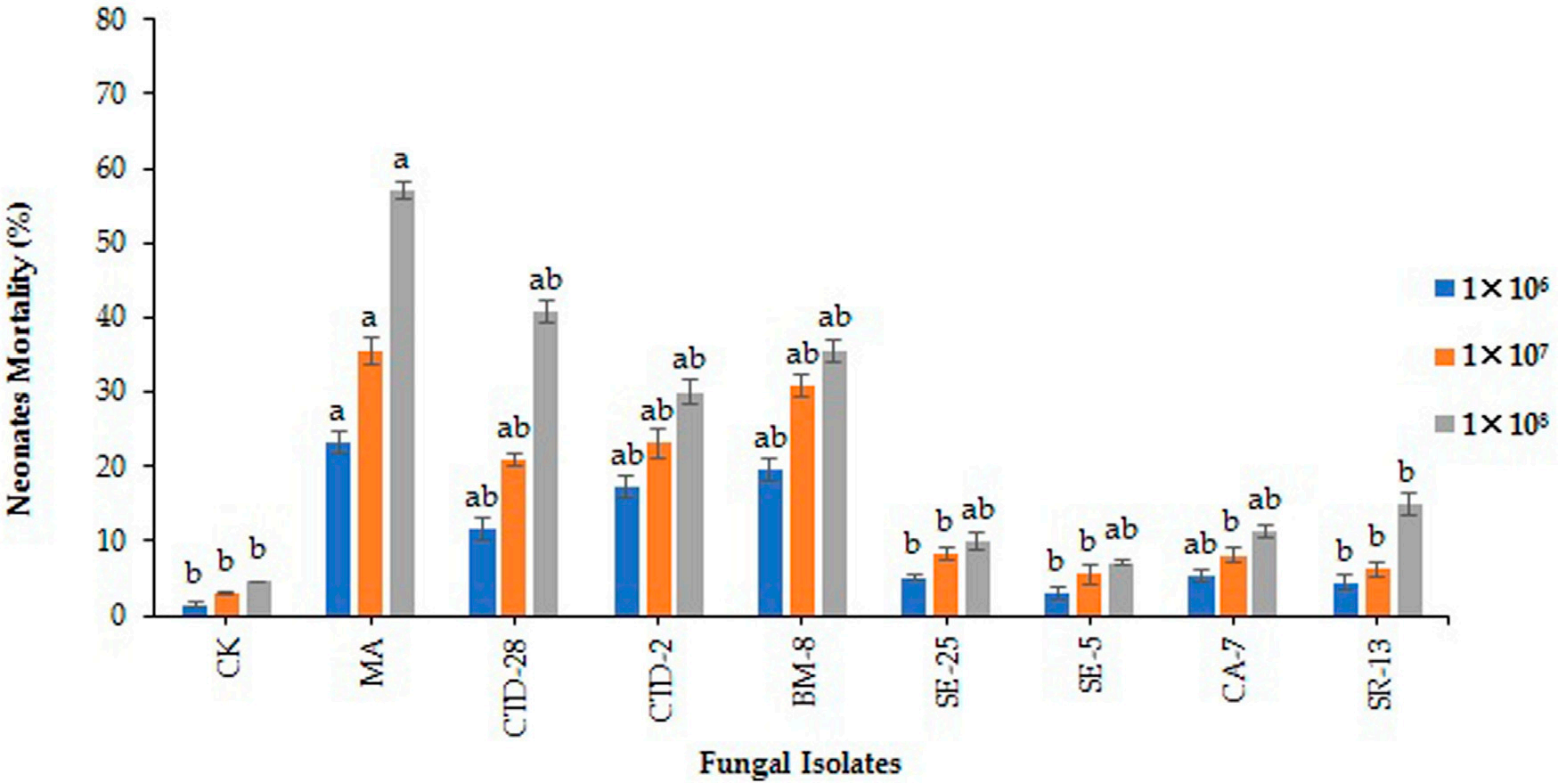
Fungal isolates induced fall armyworm’s neonate larvae mortality when treated with different concentrations at 7 days post treatment. Error bars denote the mean ± standard error at the 95% confidence interval. Means followed by the same letters are not significantly different by Tukey’s test at *p* < 0.05.

The results revealed that the *M. anisopliae* MA isolate caused 23.3% neonate larvae mortality, followed by *Cladosporium* sp. BM-8 and *Penicillium* sp. CTD-2 isolates with 19.6% and 17.1% treated with 1 × 10^6^ conidia/mL at 7 days post treatment (F_8, 18_ = 4.65; *p* = 0.0032) **(** Figure 2; Supplementary Table S2) respectively, over 3.0% in the control. *Metarhizium anisopliae* MA and *Cladosporium* sp. BM-8 isolates caused neonate larvae mortality rates of 35.6% and 30.7%, respectively, followed by *Penicillium* sp. CTD-2 and *P. citrinum* CTD-28 isolates with 23.2% and 20.9%, respectively, over the control with 1.4% treated with 1 × 10^7^ conidia/mL (F_8, 18_ = 4.24; *p* = 0.0,052) (Figure 2; Supplementary Table S3). *Metarhizium anisopliae* MA and *P. citrinum* CTD-28 isolates caused neonate mortality rates of 57.1% and 40.7%, respectively, followed by *Cladosporium* sp. BM-8 and *Penicillium* sp. CTD-2 isolates with 35.6% and 30.0%, respectively, over the control with 4.5% treated with 1 × 10^8^ conidia/mL (F_8, 18_ = 3.03; *p* = 0.0241) (Figure 2; Supplementary Table S4). Furthermore, only 5%–10% of insect cadavers showed mycosis.

### 3.3 Effect of fungal Isolates on the cumulative mortality of eggs and neonate larvae of FAW

**FIGURE 3:**
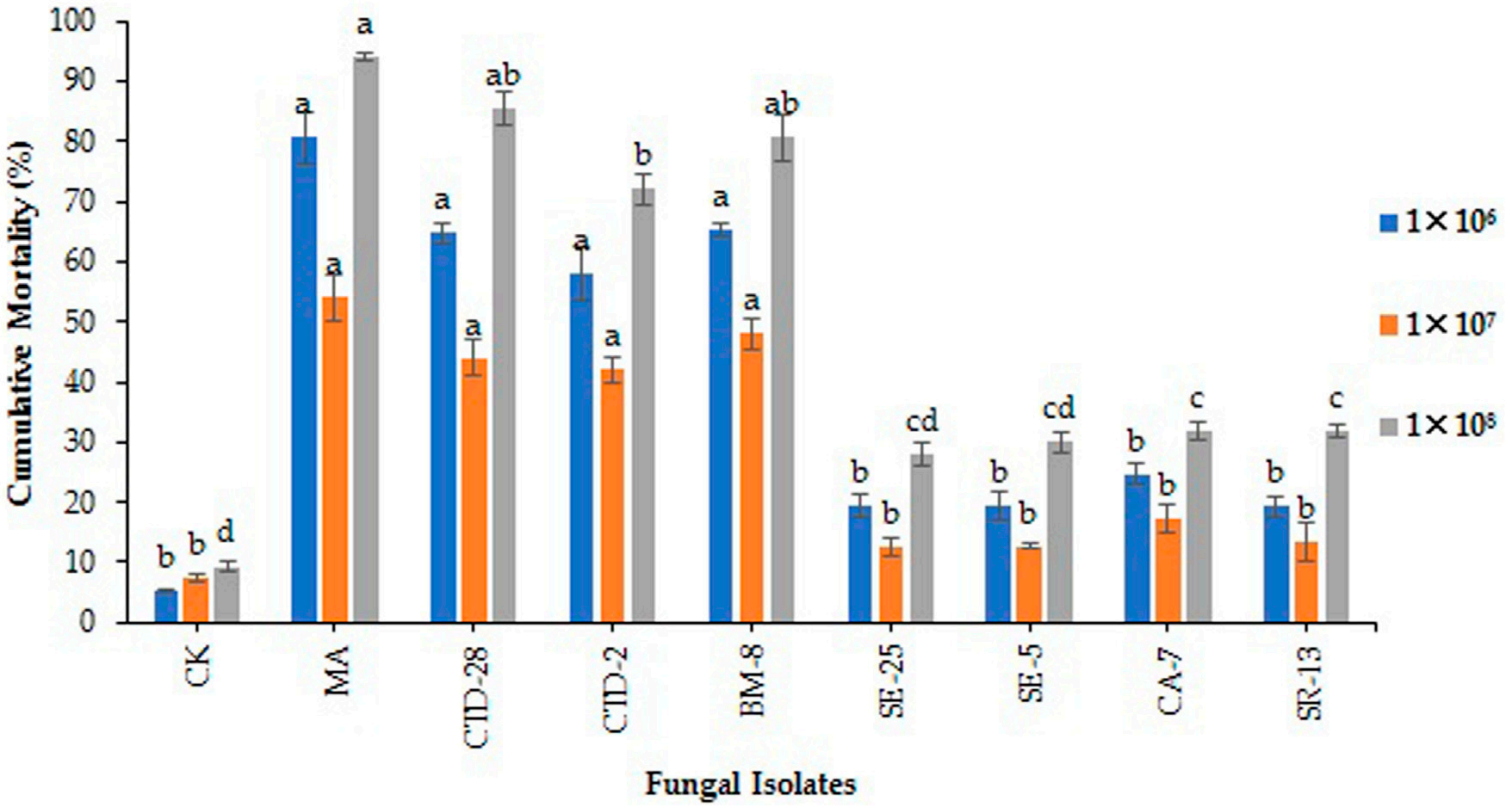
Effects of entomopathogenic fungal isolates on cumulative mortality to the eggs and neonates of fall armyworm treated with different concentrations at 14 days post treatment. Error bars denote the mean ± standard error at the 95% confidence interval. Means followed by the same letters are not significantly different by Tukey’s test at *p* < 0.05.

The results showed that *M. anisopliae* MA and *Cladosporium* sp. BM-8 isolates caused cumulative mortality of 54.0% and 48.0%, respectively, followed by *P. citrinum* CTD-28 and *Penicillium* sp. CTD-2 isolates, which caused 44.0% and 42.0% cumulative mortality to the eggs and neonates, respectively, compared to the control with 7.3%, respectively, over the control with 3.0% after treated with 1 × 10^6^ conidia/mL at14 days post treatment (F_8, 18_ = 4.7; *p* = 0.0000) (Figure 3; Supplementary Table S2). *Metarhizium anisopliae* MA, *Cladosporium* sp. BM-8 and *P. citrinum* CTD-28 isolates caused cumulative mortalities of 80.7%, 65.3%, and 64.7%, respectively, followed by *Penicillium* sp. CTD-2 with 58.0% over the control (5.3%) treated with 1 × 10^7^ conidia/mL (F_8, 18_ = 27.4; *p* = 0.0000) (Figure 3; Supplementary Table S3). *Metarhizium anisopliae* MA, *P. citrinum* CTD-28 and *Cladosporium* sp. BM-8 isolates revealed a significant effect by causing cumulative mortality of 94.0%, 85.3%, and 80.7%, respectively, followed by *Penicillium* sp. CTD-2 with 72.0% over the control (9.3%) treated with 1 × 10^8^ conidia/mL (F_8, 18_ = 52.5; *p* = 0.0000) (Figure 3; Supplementary Table S4).

### 3.4 Effect of EPF on second instar of FAW larvae

**FIGURE 4:**
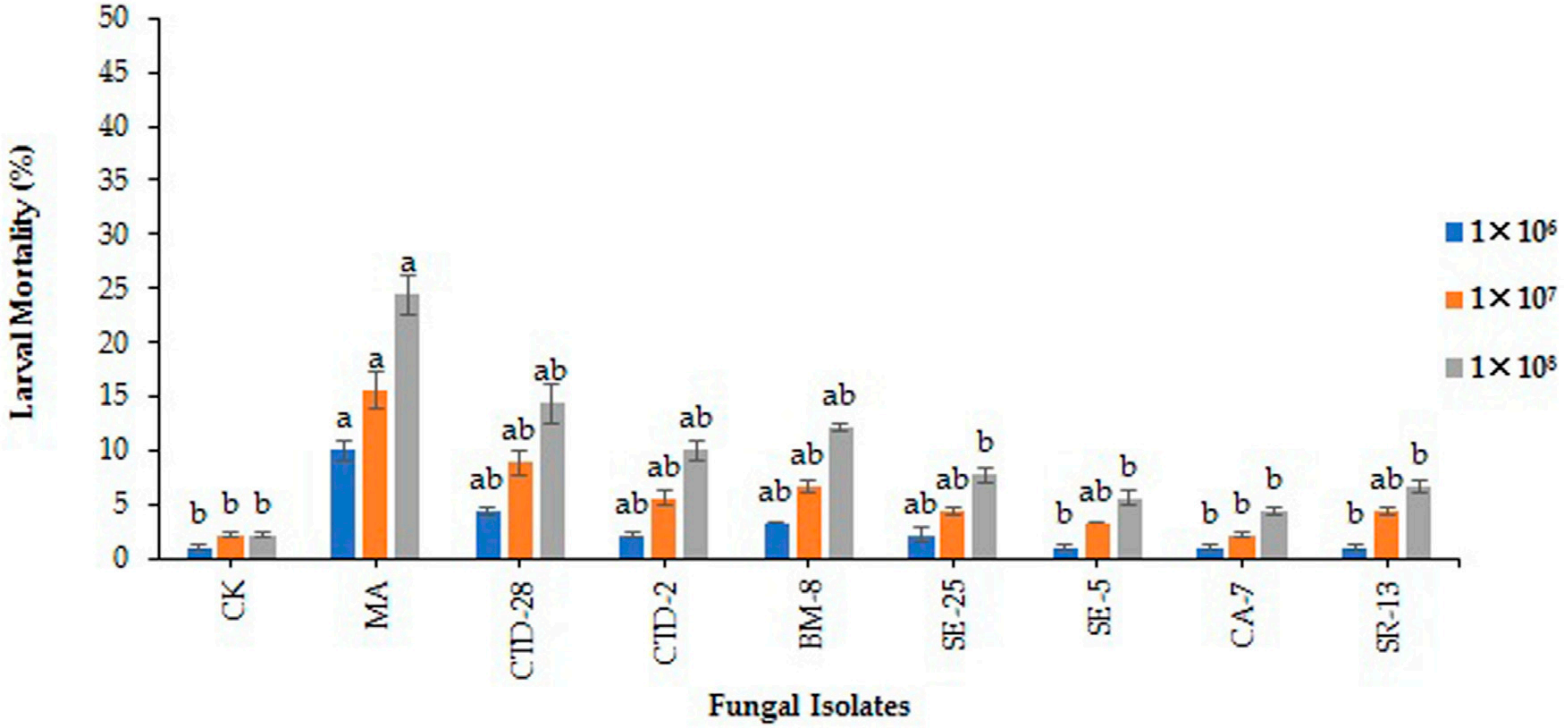
Effects of entomopathogenic fungal isolates on larval mortality of fall armyworm treated with different concentrations at 7 days post treatment. Error bars denote the mean ± standard error at the 95% confidence interval. Means followed by the same letters are not significantly different by Tukey’s test at *p* < 0.05.

The results revealed that among all tested EPF, only *Metarhizium anisopilae* MA isolates caused 10.0% larval mortality of FAW over the control (1.1%) treated with 1 × 10^6^ conidia/mL at 7 days post treatment (F_8, 18_ = 3.21; *p* = 0.0191). There was no significant difference observed by the isolates of *M. anisopilae* MA and *P. citrinum* CTD-28 over the control which caused 15.6% and 8.9% larval mortality treated with 1 × 10^7^ conidia/mL (F_8, 18_ = 2.49; *p* = 0.0514). The isolate of *M. anisopilae* MA caused 24.4% larval mortality, followed by *P. citrinum* CTD-28 with 14.4% over the control (2.21%) treated with 1 × 10^8^ conidia/mL (F_8, 18_ = 4.02; *p* = 0.0068). Furthermore, mycosis was observed only in 5% of the dead larvae with effective strains (Figure 4; Supplementary Table S5).

### 3.5 Effect of EPF on the feeding performance of second instar larvae of FAW

**FIGURE 5:**
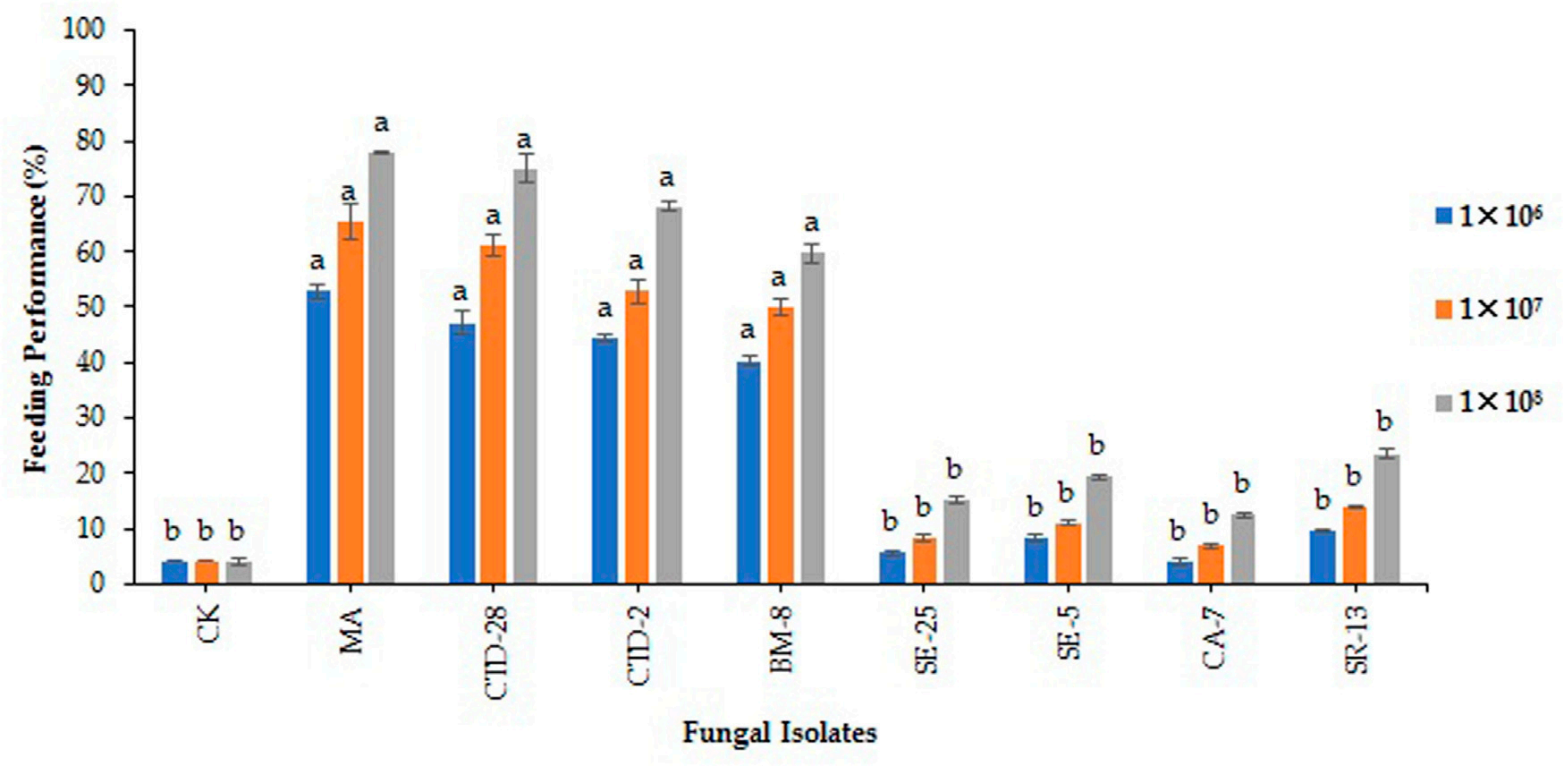
Effects of entomopathogenic fungal isolates on the feeding performance of second instar larvae of fall armyworm treated with different concentrations at 48 h post treatment. Error bars denote the mean ± standard error at the 95% confidence interval. Means followed by the same letters are not significantly different by Tukey’s test at *p* < 0.05.

The isolates of *M. anisopliae* MA and *P. citrinum* CTD-28 were found to be effective by reducing 52.8% and 47.2%, feeding performance of FAW larvae, respectively, followed by *Penicillium* sp. CTD-2 and *Cladosporium* sp. BM-8 isolates, which reduced by 44.4% and 40.3% feeding performance, respectively, over the control (4.2%) after treated with 1 × 10^6^ conidia/mL at 48 h post treatment (F_8, 18_ = 29.5; *p* = 0.0000). *Metarhizium anisopliae* MA and *P. citrinum* CTD-28 isolates significantly reduced the feeding performance by 65.3% and 61.1%, respectively, followed by *Penicillium* sp. CTD-2 and *Cladosporium* sp. BM-8 isolates, which reduced the feeding performance of FAW larvae by 52.8% and 50.0%, respectively, compared to the control (4.2%) after treated with 1 × 10^7^ conidia/mL (F_8, 18_ = 17.2; *p* = 0.0000). *Metarhizium anisopliae* MA and *P. citrinum* CTD-28 isolates outperformed all the other EPF by reducing feeding performance of 77.8% and 75.0%, respectively, followed by 68.1% and 59.7% with *Penicillium* sp. CTD-2 and *Cladosporium* sp. BM-8 isolates, respectively, over the control (4.2%) after treated with 1 × 10^8^ conidia/mL (F_8, 18_ = 38.5; *p* = 0.0000) (Figure 5; Supplementary Table S6).

### 3.6 Effect of EPF against pupae of FAW

**FIGURE 6:**
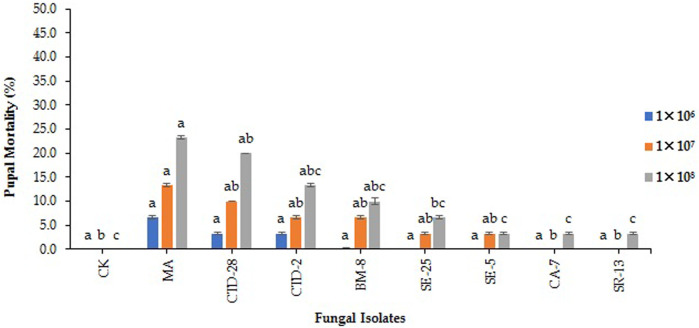
Effects of EPF on pupal mortality of fall armyworm treated with different concentrations at 14 days post treatment. Error bars denote the mean ± standard error at the 95% confidence interval. Means followed by the same letters are not significantly different by Tukey’s test at *p* < 0.05.

The results indicate that all the tested EPF isolates did not show significant pupal mortality of FAW compared to the control after treated with 1 × 10^6^ conidia/mL at 15 days posttreatment (F_8, 18_ = 1.19; *p* = 0.3591). *M. anisopilae* MA isolate caused pupal mortality of 13.3%, followed by the *P. citrinum* CTD-28 isolate with 10.0% over the control (0.0%) when treated with 1 × 10^7^ conidia/mL (F_8, 18_ = 3.65; *p* = 0.0107). *M. anisopilae* MA and *P. citrinum* CTD-28 isolates caused 23.3% and 20.0% pupal mortality, respectively, followed by *Penicillium* sp. CTD-2 and *Cladosporium* sp. BM-8 isolates with 13.3% and 10.0% compared to the control (0.0%) treated with 1× 10^8^ conidia/mL (F_8, 18_ = 5.94; *p* = 0.0008) (Figure 6; Supplementary Table S7).

### 3.7 Factorial analysis of variance

Factorial analysis of variance revealed a significant effect of the conidial concentrations (F_2, 192_ = 67.62; *p* = 0.0000), the treatments (F_7,__192_ = 59.46; *p* = 0.0000), the life stages (F_3_, _192_ = 191.46; *p* = 0.0000), and the significant interaction was observed between concentration and treatment (F_14, 192_ = 3.18; *p* = 0.0000), the concentration and life stages (F_6, 192_ = 5.65; *p* = 0.0000), the treatment and life stages (F_21, 192_ = 9.41; *p* = 0.0000), while non-significant interaction was found among concentrations, treatments and life stages (F_42, 192_ = 0.40; *p* = 0.9997) (Table 2; Supplementary Table S8).

**TABLE 2:** Mortality (Means ± SEs) of immature stages of fall armyworm treated with different concentrations of isolates of entomopathogenic fungi.

		Percent mortality ±means standard error				
Concentrations	Fungal Species	Isolates	Eggs^A^	Neonates^B^	Larvae^C^	Pupae^C^
1 × 10^6^	*M. anisopliae*	MA^A^	40.0 ± 2.5b	23.3 ± 1.5a	10.0 ± 1.0 a	6.7 ± 0.3 a
Conidia/mL^C^	*P. citrinum*	CTD-28^B^	36.7 ± 2.0a	11.6 ± 1.5ab	8.9 ± 1.2 a	3.3 ± 0.3 a
	*Penicillium* sp	CTD-2^B^	30.0 ± 1.7a	17.1 ± 1.5ab	7.8 ± 0.9 a	3.3 ± 0.3 a
	*Cladosporium* sp	BM-8^B^	35.3 ± 1.5a	19.6 ± 1.5ab	7.8 ± 0.7a	3.3 ± 0.3 a
	*A. versicolor*	SE-25^C^	8.0 ± 1.2a	5.1 ± 0.3b	4.4 ± 0.9 a	0.0 ± 0.0 a
	*Aspergillus* sp	SE-5^C^	10.0 ± 0.6b	3.0 ± 0.9b	4.4 ± 0.3 a	3.3 ± 0.3 a
	*Metarhizium* sp	CA-7^C^	12.7 ± 1.5b	5.3 ± 0.9ab	1.1 ± 0.3 a	0.0 ± 0.0 a
	*S. racemosum*	SR-13 ^C^	9.3 ± 2.2b	4.4 ± 1.0b	1.1 ± 0.3 a	0.0 ± 0.0 a
1 × 10^7^	*M. anisopliae*	MA^A^	70.0 ± 2.9a	35.6 ± 1.8a	10.0 ± 1.0 a	6.7 ± 0.3 a
Conidia/mL^B^	*P. citrinum*	CTD-28^B^	55.3 ± 1.5ab	20.9 ± 0.9ab	8.9 ± 1.2 a	3.3 ± 0.3 a
	*Penicillium* sp	CTD-2^B^	45.3 ± 2.8b	23.2 ± 2.0ab	7.8 ± 0.9 a	3.3 ± 0.3 a
	*Cladosporium* sp	BM-8^B^	50.0 ± 2.1b	30.7 ± 1.5ab	7.8 ± 0.7a	3.3 ± 0.3 a
	*A. versicolor*	SE-25^C^	12.0 ± 1.2c	8.3 ± 0.9b	4.4 ± 0.9 a	0.0 ± 0.0 a
	*Aspergillus* sp	SE-5^C^	14.7 ± 1.5c	5.5 ± 1.2b	4.4 ± 0.3 a	3.3 ± 0.3 a
	*Metarhizium* sp	CA-7^C^	18.0 ± 0.6c	8.1 ± 0.9b	1.1 ± 0.3 a	0.0 ± 0.0 a
	*S. racemosum*	SR-13^C^	14.0 ± 1.0c	6.2 ± 0.9b	1.1 ± 0.3 a	0.0 ± 0.0 a
1 × 10^8^	*M. anisopliae*	MA^A^	86.0 ± 0.6a	57.1 ± 1.2a	10.0 ± 1.0 a	6.7 ± 0.3 a
Conidia/mL^A^	*P. citrinum*	CTD-28^B^	75.3 ± 1.5ab	40.7 ± 1.5ab	8.9 ± 1.2 a	3.3 ± 0.3 a
	*Penicillium* sp	CTD-2^B^	60.0 ± 1.2c	30.0 ± 1.7ab	7.8 ± 0.9 a	3.3 ± 0.3 a
	*Cladosporium* sp	BM-8^B^	70.0 ± 2.9bc	35.6 ± 1.25ab	7.8 ± 0.7a	3.3 ± 0.3 a
	*A. versicolor*	SE-25^C^	20.0 ± 1.5de	10.0 ± 1.2ab	4.4 ± 0.9 a	0.0 ± 0.0 a
	*Aspergillus* sp	SE-5^C^	24.7 ± 1.5d	7.1 ± 0.3ab	4.4 ± 0.3 a	3.3 ± 0.3 a
	*Metarhizium* sp	CA-7^C^	23.3 ± 1.5d	11.3 ± 0.9ab	1.1 ± 0.3 a	0.0 ± 0.0 a
	*S. racemosum*	SR-13^C^	20.0 ± 0.6de	15.0 ± 1.5b	1.1 ± 0.3 a	0.0 ± 0.0 a

## 4 Discussion

There are a variety of microbial pathogens that have been associated with FAW*,* including fungi, bacteria and viruses (Gardner et al., 1984), but only a few pathogens among them are responsible for causing infection of the pests (Polanczyk et al., 2000; Garcia et al., 2008; Negrisoli et al., 2010b; Negrisoli et al., 2010a; Behle and Popham, 2012; Salvadori et al., 2012; Gómez et al., 2013). FAW nuclear polyhedrosis virus (NPV) is reported to be one of the most important pathogens inducing significant mortality to the pest. Thus, any entomopathogen that is able to cause infection to pests before it reaches its destructive stage might play a key role in the management of insect pests. Hence, the present study focused on screening selected EPF species for the control of FAW by causing infection at any susceptible stage of life.

The eggs are the most susceptible to microbial infection and require maximum nutrients for their development (Tillman, 2010); therefore, eggs are the sensitive stage by pathogenic microorganisms (Kellner, 2002; Trougakos and Margaritis, 2002). The results of the present study revealed that FAW eggs were the most vulnerable to the tested fungal isolates. Our results are supported by previous studies where isolates of *M. anisopliae* and *Cladosporium tenuissimum* showed 96.5% and 55.6% FAW egg mortality (Akutse et al., 2019; Idrees et al., 2021). The isolate of *Cladosporium sp.* was significantly virulent against *Helicoverpa armigera* egg mortality (Bahar et al., 2011). The findings of our study are in line with those of Idrees et al. (Idrees et al., 2021), who observed that isolates of *Aspergillus* sp. did not induce FAW egg mortality. The highest egg mortality of *Spodoptera litura* was observed by Anand and Tiwary (2009), when treated with isolates of *Aspergillus* sp. Similar to our results, the highest egg mortality of FAW was observed with the isolates of *P. citrinum* and *Pteroptyx bearni* (Foo et al., 2017; Idrees et al., 2021). Histopathological research proved that fungal spores can successfully penetrate eggs and cause fungal infections (Pires et al., 2009; Zhang et al., 2014).

The results of our research are supported by previous findings where isolates of *M. anisopliae* were significantly effective in inducing FAW neonate mortality (Akutse et al., 2019), while the isolates of *Aspergillus* sp, *C. tenuissimum* and *P. citrimum* did not induce significant mortality against neonates of FAW (Idress et al., 2021). The isolates of *P. citrinum* and *C. tenuissimum* caused significant cumulative mortality to the eggs and neonates of FAW in the present research. The isolates of *Cladosporium aphidis* were significantly effective in causing cumulative mortality of aphid species (Gui et al., 2005). Although all host stages are not equally susceptible to pathogen infection, EPF have the potential to cause infection of insect pests at any stage of their life (Opisa et al., 2018).

The tested EPF were not effective against second instar larvae of FAW. The isolates of *Aspergillus* sp. did not cause significant larval mortality in second instar larvae of FAW and *Chilo suppressalis* (Idrees et al., 2021; Shahriari et al., 2021). The isolate of *P. citrinum* was found to be the most effective against second instar larvae of *S. litura* but ineffective in causing larval mortality of FAW (Herlinda et al., 2020). The isolates of *P. citrinum* were found to be associated with mosquito larvae (Da et al., 2009) and caused significant larval mortality against *Culex quinquefasciatus* (Maketon et al., 2014). Interestingly, it was observed that some of the fungal isolates showed significant effectiveness against early instar larvae, while less virulent to mature larvae; for example, the isolate of *Cladosporium* sp. caused significant mortality of early instar larvae compared with matured larvae of *H. armigera* (Bahar et al., 2011). Fewer species in the *Cladosporium* genus were found to be virulent against aphids and whiteflies (Abdel-Baky and Abdel-Salam, 2003; Gui et al., 2005). The isolates of *M. anisopliae* did not show significant mortality against second instar larvae of FAW (Akutse et al., 2019). The effectiveness level of each fungal isolate varies for causing infection against FAW (Fargues and Maniania, 1992). Therefore, this situation provides a basis for further studies to reveal the mechanisms of higher resistance in FAW larvae to the tested fungal isolates in this study. The high resistance of the mature larvae against fungal infection may be due to the larval integument that does not permit effective penetration of the fungal spore (Bosa et al., 2004). There are minimal chances of fungal infection when the inoculum is lost due to molting, even though molting does not constantly result in the prevention of fungal infection (Meekes, 2001). The low susceptibility of larvae in the present study could be a feature of the tested fungal isolates and might be attributed to genetic diversity or perhaps phenotypic differences between the populations of FAW (Monnerat et al., 2006).

The production of toxic substances by EPF inside the host body leads to mechanical disruption in insect structural integrity, which ultimately reduces the feeding performance of insect pests (Tefera and Pringle, 2003). A previous study reported a significant feeding reduction in the feeding performance of insect pests treated with different fungal isolates (Fargues et al., 1994; Ekesi and Maniania, 2000; Hussain et al., 2009; Migiro et al., 2011; Idrees et al., 2021; Idrees et al., 2022a). The reduction in feeding performance by larvae treated with fungal isolates is one of the key factors in host mortality, indicating virulence of fungal isolates, and requires further investigation to assess the level of pathogenicity or antifeedant effects (Ondiaka et al., 2008).

The previous research finding concluded that EPF did not infect to all the stages of lepidopteran pests equally and in most of the research finds have been observed that EPF have to potential to infect the earliest stages of pest effective compared with later stages (Idrees et al., 2022b). It might be the reason that the resistance developed in later mature stages of pest. The fungal isolates did not show a significant effect in causing pupal mortality of FAW in our research. The previous findings are consistent with our study results, where fungal isolates did not cause significant pupal mortality of *S. litura* within 14 days post treatment (Anand et al., 2009; Asi et al., 2013). The different life stages of any insect do not response to any pathogen stress the same way. So, it is ‘very normal’ to test EPF on various life stages in order to understand which life stage is most susceptible, or either way, the most resistant to the said fungal isolate. As this would inform users the most appropriate stage of the insect to target for effective biological control programs as in our previous study concluded that eggs are the most susceptible stage for target as compared to the other life stages of FAW (Aktuse et al., 2019; Idrees et al., 2021).

## 5 Conclusion

The *M. anisopliae*, *P. citrinum*, *Penicillium* sp. and *Cladosporium* sp. have potential to infect the immature stages and feeding performance of FAW. Therefore, these EPF could be considered for the development of microbial pesticides against FAW. Further studies are needed to insight the key toxins which are responsible for affecting the physiological function of FAW and slowing down the feeding performance.

## Data Availability

The original contributions presented in the study are included in the article/Supplementary Material, further inquiries can be directed to the corresponding author.
