# Supplementary material for: Virulence of entomopathogenic fungi against fall armyworm, Spodoptera frugiperda (Lepidoptera: Noctuidae) under laboratory conditions
Source: Front Physiol. 2023 Mar 8;14:1107434. doi: 10.3389/fphys.2023.1107434 (PMC10031024; doi:10.3389/fphys.2023.1107434)
Supplement: Supplementary file 1 [file DataSheet1.docx]

**Supplementary Table S1.** Germination rates of the entomopathogenic fungal isolates used in the study against second instar larvae of fall armyworm

|  | Germination (%) ± SE | | | |
| --- | --- | --- | --- | --- |
| Fungal Species | Isolates | 1 x 10^6^  Conidia/mL | 1 x 10^7^  Conidia/mL | 1 x 10^8^  Conidia/mL |
| *M. anisopliae* | MA | 90.7 ± 3.0 | 90.0 ± 2.0 | 90.0 ± 1.5 |
| *P. citrinum* | CTD-28 | 90.0 ± 0.6 | 89.3 ± 1.2 | 90.3 ± 1.9 |
| *Penicillium* sp. | CTD-2 | 89.0 ± 0.6 | 90.3 ± 0.3 | 90.0 ± 1.2 |
| *Cladosporium* sp. | BM-8 | 90.3 ± 1.2 | 91.0 ± 0.6 | 88.0 ± 2.6 |
| *A. versicolor* | SE-25 | 88.7 ± 1.9 | 89.0 ± 0.6 | 89.3 ± 1.5 |
| *Aspergillus* sp. | SE-5 | 88.0 ± 2.0 | 88.3 ± 1.5 | 88.3 ± 2.6 |
| *Metarhizium* sp. | CA-7 | 88.0 ± 2.0 | 88.3 ± 1.2 | 88.0 ± 1.0 |
| *S. racemosum* | SR-13 | 89.7 ± 1.5 | 88.7 ± 0.9 | 88.9 ± 1.8 |

|  | Mortality (%) ± SE | | | |
| --- | --- | --- | --- | --- |
| Fungal Species | Isolates | Eggs  Mortality | Neonates Mortality | Cumulative  Mortality |
| *M. anisopliae* | MA | 40.0 ± 2.5 a | 23.3 ± 1.5 a | 54.0 ± 4.0 a |
| *P. citrinum* | CTD-28 | 36.7 ± 2.0 a | 11.6 ± 1.5 ab | 44.0 ± 3.1 a |
| *Penicillium* sp. | CTD-2 | 30.0 ± 1.7 a | 17.1 ± 1.5 ab | 42.0 ± 2.1 a |
| *Cladosporium* sp. | BM-8 | 35.3 ± 1.5 a | 19.6 ± 1.5 ab | 48.0 ± 2.5 a |
| *A. versicolor* | SE-25 | 8.0 ± 1.2 b | 5.1 ± 0.3 b | 12.7 ± 1.5 b |
| *Aspergillus* sp. | SE-5 | 10.0 ± 0.6 b | 3.0 ± 0.9 b | 12.7 ± 0.3 b |
| *Metarhizium* sp. | CA-7 | 12.7 ± 1.5 b | 5.3 ± 0.9 ab | 17.3 ± 2.3 b |
| *S. racemosum* | SR-13 | 9.3 ± 2.2 b | 4.4 ± 1.0 b | 13.3 ± 3.2 b |
| *Control* |  | 6.0 ± 0.6 c | 3.0 ± 0.3 b | 7.3 ± 0.7 b |
| *F* |  | 19.3 | 4.65 | 14.7 |
| *df* |  | 8 | 8 | 8 |
| *P* |  | 0.000 | 0.003 | 0.000 |

The values within column represent mean ± standard error.

**Supplementary Table S2.** Effect of entomopathogenic fungal isolates on cumulative mortality of eggs and neonate larvae of fall armyworm treated with 1 × 10^6^ conidia/ml.

Means ± standard error within a column not sharing common letters are significantly different by Tukey’s test at *p* < 0.05.

**Supplementary Table S3.** Effect of entomopathogenic fungal isolates on cumulative mortality of eggs and neonate larvae of fall armyworm treated with 1 × 10^7^ conidia/ml.

|  | Mortality (%) ± SE | | | |
| --- | --- | --- | --- | --- |
| Fungal Species | Isolates | Eggs  Mortality | Neonates  Mortality | Cumulative  Mortality |
| *M. anisopliae* | MA | 70.0 ± 2.9 a | 35.6 ± 1.8 a | 80.7 ± 4.3 a |
| *P. citrinum* | CTD-28 | 55.3 ± 1.5 ab | 20.9 ± 0.9 ab | 64.7 ± 1.9 a |
| *Penicillium* sp. | CTD-2 | 45.3 ± 2.8 b | 23.2 ± 2.0 ab | 58.0 ± 4.6 a |
| *Cladosporium* sp. | BM-8 | 50.0 ± 2.1 b | 30.7 ± 1.5 ab | 65.3 ± 0.9 a |
| *A. versicolor* | SE-25 | 12.0 ± 1.2 c | 8.3 ± 0.9 b | 19.3 ± 2.0 b |
| *Aspergillus* sp. | SE-5 | 14.7 ± 1.5 c | 5.5 ± 1.2 b | 19.3 ± 2.3 b |
| *Metarhizium* sp. | CA-7 | 18.0 ± 0.6 c | 8.1 ± 0.9 b | 24.7 ± 1.8 b |
| *S. racemosum* | SR-13 | 14.0 ± 1.0 c | 6.2 ± 0.9 b | 19.3 ± 1.8 b |
| *Control* |  | 4.0 ± 0.6 b | 1.4 ± 0.3 b | 5.3 ± 0.3 b |
| *F* |  | 44.1 | 4.24 | 27.4 |
| *df* |  | 8 | 8 | 8 |
| *P* |  | 0.000 | 0.005 | 0.000 |

Means ± standard error within a column not sharing common letters are significantly different by Tukey’s test at *p* < 0.05.

**Supplementary Table S4.** Effect of entomopathogenic fungal isolates on cumulative mortality of eggs and neonate larvae of fall armyworm treated with 1 × 10^8^ conidia/ml.

|  | Mortality (%) ± SE | | | |
| --- | --- | --- | --- | --- |
| Fungal Species | Isolates | Eggs  Mortality | Neonates Mortality | Cumulative  Mortality |
| *M. anisopliae* | MA | 86.0 ± 0.6 a | 57.1 ± 1.2 a | 94.0 ± 0.6 a |
| *P. citrinum* | CTD-28 | 75.3 ± 1.5 ab | 40.7 ± 1.5 ab | 85.3 ± 2.8 a |
| *Penicillium* sp. | CTD-2 | 60.0 ± 1.2 c | 30.0 ± 1.7 ab | 72.0 ± 2.5 a |
| *Cladosporium* sp. | BM-8 | 70.0 ± 2.9 bc | 35.6 ± 1.25 ab | 80.7 ± 3.9 b |
| *A. versicolor* | SE-25 | 20.0 ± 1.5 de | 10.0 ± 1.2 ab | 28.0 ± 2.1 bc |
| *Aspergillus* sp. | SE-5 | 24.7 ± 1.5 d | 7.1 ± 0.3 ab | 30.0 ± 1.7 de |
| *Metarhizium* sp. | CA-7 | 23.3 ± 1.5 d | 11.3 ± 0.9 ab | 32.0 ± 1.5 cd |
| *S. racemosum* | SR-13 | 20.0 ± 0.6 de | 15.0 ± 1.5 b | 32.0 ± 1.0 ef |
| *Control* |  | 7.3 ± 0.9 e | 4.5 ± 0.0 b | 9.3 ± 0.9 d |
| *F* |  | 99.1 | 3.03 | 52.5 |
| *df* |  | 8 | 8 | 8 |
| *P* |  | 0.000 | 0.024 | 0.000 |

Means ± standard error within a column not sharing common letters are significantly different by Tukey’s test at *p* < 0.05.

**Supplementary Table S5.** Effects of entomopathogenic fungal isolates on mortality of the second instar larvae of fall armyworm treated with different concentrations.

Means ± standard error within a column not sharing common letters are significantly different by Tukey’s test at *p* < 0.05.

|  | Larval Mortality (%) ± SE | | | |
| --- | --- | --- | --- | --- |
| Fungal Species | Isolates | 1 x 10^6^  conidia/mL | 1 x 10^7^  conidia/mL | 1 x 10^8^  conidia/mL |
| *M. anisopliae* | MA | 10.0 ± 1.0 a | 15.6 ± 1.8 a | 24.4 ± 1.9 a |
| *P. citrinum* | CTD-28 | 4.4 ± 0.3 ab | 8.9 ± 1.2 ab | 14.4 ± 1.8 ab |
| *Penicillium* sp. | CTD-2 | 2.2 ± 0.3 ab | 5.6 ± 0.7 ab | 10.0 ± 1.0 ab |
| *Cladosporium* sp. | BM-8 | 3.3 ± 0.0 ab | 6.7 ± 0.6 ab | 12.2 ± 0.3 ab |
| *A. versicolor* | SE-25 | 2.2 ± 0.7 ab | 4.4 ± 0.3 ab | 7.8 ± 0.7 b |
| *Aspergillus* sp. | SE-5 | 1.1 ± 0.3 b | 3.3 ± 0.0 b | 5.6 ± 0.7 b |
| *Metarhizium* sp. | CA-7 | 1.1 ± 0.3 b | 2.2 ± 0.3 b | 4.4 ± 0.3 b |
| *S. racemosum* | SR-13 | 1.1 ± 0.3 b | 4.4 ± 0.3 ab | 6.7 ± 0.6 b |
| *Control* |  | 1.1 ± 0.3 b | 2.2 ± 0.3 b | 2.2 ± 0.3 b |
| *F* |  | 3.21 | 2.49 | 4.02 |
| *df* |  | 8 | 8 | 8 |
| *P* |  | 0.019 | 0.051 | 0.006 |

**Supplementary Table S6.** Effects of entomopathogenic fungal isolates on feeding performance of the second instar larvae of fall armyworm treated with different concentrations.

|  | Feeding Efficacy (%) ± SE | | | |
| --- | --- | --- | --- | --- |
| Fungal Species | Isolates | 1 x 10^6^  conidia/mL | 1 x 10^7^  conidia/mL | 1 x 10^8^  conidia/mL |
| *M. anisopliae* | MA | 52.8 ± 1.2 a | 65.3 ± 3.2 a | 77.8 ± 0.3 a |
| *P. citrinum* | CTD-28 | 47.2 ± 2.0 a | 61.1 ± 1.8 a | 75.0 ± 2.5 a |
| *Penicillium* sp. | CTD-2 | 44.4 ± 0.7 a | 52.8 ± 2.0 a | 68.1 ± 0.9 a |
| *Cladosporium* sp. | BM-8 | 40.3 ± 0.9 a | 50.0 ± 1.5 a | 59.7 ± 1.8 a |
| *A. versicolor* | SE-25 | 5.6 ± 0.3 b | 8.3 ± 0.6 b | 15.3 ± 0.7 b |
| *Aspergillus* sp. | SE-5 | 8.3 ± 0.6 b | 11.1 ± 0.3 b | 19.4 ± 0.3 b |
| *Metarhizium* sp. | CA-7 | 4.2 ± 0.6 b | 6.9 ± 0.3 b | 12.5 ± 0.6 b |
| *S. racemosum* | SR-13 | 9.7 ± 0.3 b | 13.9 ± 0.3 b | 23.6 ± 0.7 b |
| *Control* |  | 4.2 ± 0.0 b | 4.2 ± 0.0 b | 4.2 ± 0.6 b |
| *F* |  | 29.5 | 17.2 | 38.5 |
| *df* |  | 8 | 8 | 8 |
| *P* |  | 0.000 | 0.000 | 0.000 |

Means ± standard error within a column not sharing common letters are significantly different by Tukey’s test at p < 0.05.

**Supplementary Table S7.** Effect of entomopathogenic fungal isolates on pupal mortalities of fall armyworm treated with different concentrations.

|  | Pupal Mortality (%) ± SE | | | |
| --- | --- | --- | --- | --- |
| Fungal Species | Isolates | 1 x 10^6^  conidia/mL | 1 x 10^7^  conidia/mL | 1 x 10^8^  conidia/mL |
| *M. anisopliae* | MA | 6.7 ± 0.3 a | 13.3 ± 0.3 a | 23.3 ± 0.3 a |
| *P. citrinum* | CTD-28 | 3.3 ± 0.3 a | 10.0 ± 0.0 ab | 20.0 ± 0.0 ab |
| *Penicillium* sp. | CTD-2 | 3.3 ± 0.3 a | 6.7 ± 0.3 ab | 13.3 ± 0.3 abc |
| *Cladosporium* sp. | BM-8 | 3.3 ± 0.3 a | 6.7 ± 0.3 ab | 10.0 ± 0.6 abc |
| *A. versicolor* | SE-25 | 0.0 ± 0.0 a | 3.3 ± 0.3 ab | 6.7 ± 0.3 bc |
| *Aspergillus* sp. | SE-5 | 0.0 ± 0.0 a | 3.3 ± 0.3 ab | 3.3 ± 0.3 c |
| *Metarhizium* sp. | CA-7 | 0.0 ± 0.0 a | 0.0 ± 0.0 b | 3.3 ± 0.3 c |
| *S. racemosum* | SR-13 | 0.0 ± 0.0 a | 0.0 ± 0.0 b | 3.3 ± 0.3 c |
| *Control* |  | 0.0 ± 0.0 a | 0.0 ± 0.0 b | 0.0 ± 0.0 c |
| *F* |  | 1.19 | 3.65 | 5.94 |
| *df* |  | 8 | 8 | 8 |
| *P* |  | 0.359 | 0.010 | 0.000 |

Means ± standard error within a column not sharing common letters are significantly different by Tukey’s test at p < 0.05.

**Supplementary Table S8.** Analysis of variance comparison table for mean percent mortality of eggs, neonate, larvae and pupae of fall armyworm treated with different concentrations of isolates of *B. bassiana* under laboratory conditions.

| Source | DF | SS | MS | F | P |
| --- | --- | --- | --- | --- | --- |
| Concentrations | 2 | 9369 | 4684.4 | 67.62 | 0.0000 |
| Treatments | 7 | 28831 | 4118.7 | 59.46 | 0.0000 |
| Life Stages | 3 | 39789 | 13262.9 | 191.46 | 0.0000 |
| Conc*Treat | 14 | 3085 | 220.4 | 3.18 | 0.0000 |
| Conc*Stages | 6 | 2349 | 391.5 | 5.65 | 0.0000 |
| Treat*Stages | 21 | 13689 | 651.9 | 9.41 | 0.0000 |
| Conc*Treat*Stages | 42 | 1157 | 27.6 | 0.40 | 0.9997 |
| Error | 192 | 13300 | 69.3 |  |  |
| Total | 287 | 111569 |  |  |  |
| Grand Mean | 16.549 |  |  |  |  |
| CV | 50.29 |  |  |  |  |
